# Withanolide D induces apoptosis in leukemia by targeting the activation of neutral sphingomyelinase-ceramide cascade mediated by synergistic activation of c-Jun N-terminal kinase and p38 mitogen-activated protein kinase

**DOI:** 10.1186/1476-4598-9-239

**Published:** 2010-09-13

**Authors:** Susmita Mondal, Chandan Mandal, Rajender Sangwan, Sarmila Chandra, Chitra Mandal

**Affiliations:** 1 Infectious diseases and immunology Division, Indian Institute of Chemical Biology, A Unit of Council of Scientific and Industrial Research, Govt. of India; 4, Raja S. C. Mullick Road, Kolkata 700032, India; 2 Central Institute of Medicinal and Aromatic Plants, Lucknow-226015, India; 3 Kothari Medical Center, 8/3, Alipore Road, Kolkata 700027, India; 4 Current Address: Department of Microbiology, Sammilani Mahavidyalaya, Baghajatin, E.M By Pass, Kolkata-700075, India

## Abstract

**Background:**

Ceramide is an important second messenger that has diverse cellular and biological effect. It is a specific and potent inducer of apoptosis and suppressor of cell growth. In leukemia, chemoresistance generally developed due to deregulated ceramide metabolism. In combinatorial treatment strategies of leukemia, few components have the capability to increases ceramide production. Manipulation in ceramide production by physiological and pharmacological modulators therefore will give additive effect in leukemia chemotherapy.

**Results:**

Here, we show that Withanolide D (C_4_β-C_5_β,C_6_β-epoxy-1-oxo-,20β, dihydroxy-20*S*,22*R*-witha-2,24-dienolide; WithaD), a pure herbal compound isolated from *Withania somnifera *could effectively induces apoptosis in a dose and time dependant manner both in myeloid (K562) and lymphoid (MOLT-4) cells being nontoxic to normal lymphocytes and control proliferative cells. WithaD potentially augment ceramide production in these cells. Downstream of ceramide, WithaD acted on MKK group of proteins and significantly increased JNK and p38MAPK phosphorylation. Pharmacological inhibition of p38MAPK and JNK proves their cooperative action on WithaD-induced cell death. Dissecting the cause of ceramide production, we found activation of neutral sphingomyelinase and showed neutral-sphingomyelinase 2 (N-SMase 2) is a critical mediator of WithaD-induced apoptosis. Knockdown of N-SMase 2 by siRNA and inhibitor of N-SMase (GW4869) significantly reduced WithaD-induced ceramide generation and phosphorylation of MKK4 and MKK3/6, whereas phosphorylation of MKK7 was moderately regulated in leukemic cells. Also, both by silencing of N-SMase 2 and/or blocking by GW4869 protects these cells from WithaD-mediated death and suppressed apoptosis, whereas Fumonisin B1, an inhibitor of ceramide synthase, did not have any effect. Additionally, WithaD effectively induced apoptosis in freshly isolated lymphoblasts from patients and the potent cell killing activity was through JNK and p38MAPK activation.

**Conclusion:**

Our results demonstrate that WithaD enhance the ceramide accumulation by activating N-SMase 2, modulate phosphorylation of the JNK and p38MAPK and induced apoptosis in both myeloid and lymphoid cells along with primary cells derived from leukemia patients. Taken together, this pure herbal compound (WithaD) may consider as a potential alternative tool with additive effects in conjunction with traditional chemotherapeutic treatment, thereby accelerate the process of conventional drug development.

## Introduction

Apoptosis is a regulated biochemical process that balance between cell survival and death, maintaining the normal tissue homeostasis [1]. In the molecular event of apoptosis, it has been thought that mainly kinases and caspases play the central role by mediating and transducing signals, but emerging reports showed that lipid molecules also play a crucial role. Different kinds of lipids reside in cell membrane and they could be released and transduce a signal from extracellular stimuli [2,3]. Among them, sphingolipid ceramide is a key lipid second messenger that regulates diverse cellular processes like cell cycle arrest, cell death, differentiation, ageing and immune response [4]. In fact, during the apoptosis, the concomitant ceramide formation from sphingomyelin hydrolysis brings the changes in membrane topology, which is the hallmark of apoptosis [5]. Ceramide regulates diverse stress signaling pathways by affecting transcription (through c-Jun), translation (through RAX), and the apoptotic machinery in many ways. Additionally, survival pathways mediated by PKC and Akt were inactivated by ceramide [6]. The importance of ceramide regulated diverse signaling pathways and its responsibility in apoptosis is therefore obvious and manifold. Hence, defects in ceramide metabolism potentially affect cellular responses to the chemotherapy or other anti-cancer strategies, making the cells more resistant and contribute to the multi drug resistance [7]. This information advocates the candidature of ceramide as a potent drug target and implies its role towards the response against malignancy.

Leukemia is heterogeneous group of neoplasm arising from the malignant transformation of haematopoietic cells [8-11]. In lymphoid leukemia, though 80% of patients achieved clinical remission in the Western countries, but in developing and under developed countries the scenario is quiet different. The problems still persist, as relapse rate is very high due to the presence of non-detectable yet existing leukemic cell mass known as minimal residual disease [12-14]. Another major problem is the development of multi drug resistance in these patients [15]. Obeid et al [16], in their classic paper reported that in leukemia, C2-ceramide, a synthetic ceramide analog is capable of inducing DNA fragmentation. Interestingly, resistance to radiation therapy developed due to defective ceramide metabolism was reported in Burkitt's lymphoma and myeloid leukemia [17]. Thus, deregulation of ceramide production may play an important role in chemoresistance. Any alteration in ceramide metabolism is often harmful to leukemia patient due to less ceramide deposition. Thereby manipulation of ceramide metabolism in patients to promote ceramide production may be helpful in chemotherapeutic treatment [18]. Both in irradiation and chemotherapeutic drug treatment strategy, the common components encourage ceramide production in leukemia [19-21]. Hence, a novel compound that could augment the production of ceramide during chemotherapy, potentiating the cell killing and leading to more effective anti-leukemic strategies, is on demand.

Withanolide D (WithaD) is a pure herbal compound purified from the ancient medicinal plant *Withania somnifera *[22]. We have recently demonstrated that WithaD induced apoptosis in both myeloid (K562) and lymphoid (MOLT-4) cells and suppress tumor cell growth in K562 xenograft (personal communication). Here, we observed that WithaD could enhance the ceramide accumulation by activating neutral-sphingomyelinase (N-SMase) and thus modulate the phosphorylation of stress kinases, JNK and p38MAPK leading to apoptosis in these leukemic cells and primary cells derived from leukemia patients. Knockdown of N-SMase 2 by siRNA and inhibitor of N-SMase (GW4869) significantly reduced WithaD-induced ceramide generation and regulated phosphorylation of MKK4 and MKK3/6 and MKK7. To the best of our knowledge, there was no study so far exploit the insight of sphingomyelinase-ceramide cascade as a result of WithaD treatment by demonstrating N-SMase 2 as a critical mediator of WithaD-induced apoptosis.

## Materials and methods

### Withanolide D

**Figure 1:**
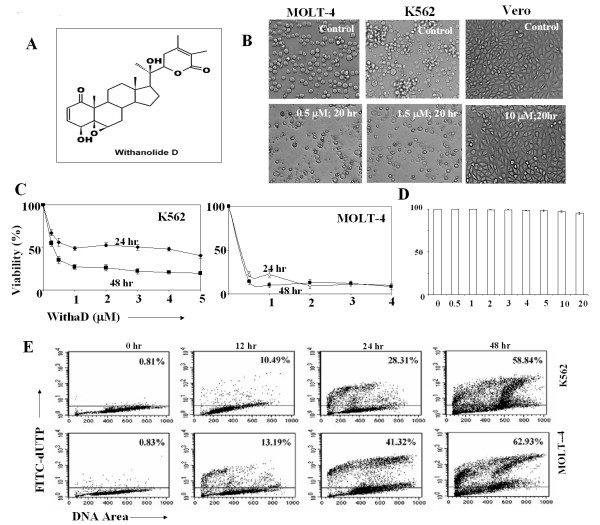
**Anti-proliferative effect of WithaD**. (A) Chemical structure of WithaD. (B) Morphological changes induced by WithaD in K562, MOLT-4 and Vero cells, as demonstrated by phase contrast microscopy. (C) Effect of WithaD (0-5 μM) on the cell viability of K562 and MOLT-4 cells after 24 and 48 hr as demonstrated by MTT assay. (D) Normal lymphocytes were isolated form normal blood, cultured for 48 hr in the presence of WithaD at concentration ranged from 0-20 μM and checked the cell viability by trypan blue dye exclusion assay. (E) Flow cytometric analysis revealed *in situ *DNA fragmentation by TUNEL assay in WithaD treated MOLT-4 (0.5 μM) and K562 (1.5 μM) cells at indicated time. The plots are representative of three independent experiments. The percentages of TUNEL positive cells were indicated in respective panel.

WithaD (M.W 470.6) was purified in high yields from Withania somnifera (Chemotype NMITLI-135, patent submitted) leaves as described previously [22]. The pure compound was crystallized and analyzed by IR, mass, ^1^H-NMR and ^13^C-NMR spectral analysis [Additional file 1]. The chemical structure of WithaD is C4β-hydroxyC5β,C6β-epoxy-1-oxo-,C20β,dihydroxy-20S,22R-witha-2,24-dienolide (Figure 1A). It was dissolved in absolute ethanol as 0.5 mM solution and stored at -70°C.

### Reagents

Recombinant E. coli diacylglycerol (DAG) kinase, 3-(4, 5-dimethylthiazol-2-yl)-2, 5-diphenyl tetrazolium bromide (MTT), anti-ceramide antibody, standard ceramide and sphingomyelin were from Sigma (St Louis, MO). APO-DIRECT kit, Bcl2, Bax, caspase 3 antibodies and 7-aminoactinomycin D (7-AAD) were from BD (San Diego, CA, USA). Cocktail protease inhibitor, inhibitors of N-SMase (GW4869), ceramide synthase (Fumonisin B1), JNK (SP600125) and p38MAPK (SB203580) were purchased from Calbiochem. Phospho-SAPK/JNK pathway sampler kit, phospho-p38MAPK pathway sampler kit, anti-phospho-MKK7, anti-MKK7, anti-phospho-SEK1/MKK4, anti-SEK1/MKK4, anti-MKK3, anti-JNK, anti-p38, anti-ERK, anti-phospho-ERK, β-actin were from Cell Signaling technology. Custom primers, siRNAs, PCR reagent kit, RNase free DNase I and Amplex Red sphingomyelinase assay kit, Opti-MEM™, Lipofectamine 2000 were purchased from Invitrogen (USA).

### Clinical samples, cell lines and culture conditions

The study involved fresh leukemia cells obtained from clinically confirmed leukemia patients (n = 22) comprises pediatric myeloid (n = 7) leukemia, B-(n = 10) and T-(n = 5) ALL, enriched with Ficoll gradient centrifugation. The diagnosis was established on the basis of morphological examination and immunophenotyping by four colour FACS analysis using commercially available lineage specific standard antibodies whose expression are known to aberrant in leukemia [9,13]. The clinical samples were collected at Kothari Medical Center and sent to the Indian Institute of Chemical Biology. Normal healthy individuals (n = 7) of both sexes served as controls. The Institutional human ethical committee had approved the study and samples were taken with the consent of the donors, patients, or their parents or guardians.

A representative cell line from chronic myeloid (K562) and T-lineage acute lymphoid origin (MOLT-4) were obtained from American Type Culture Collection (ATCC, Manassas, VA). Cells were cultured in RPMI-1640 medium supplemented with 10% fetal bovine serum (FBS; Gibco/BRL, NY, USA) in 5% CO_2_-95% air humidified atmosphere at 37°C.

### MTT assay and Trypan blue dye exclusion assay

Cytotoxicity of WithaD against MOLT-4, K562 and primary cells from leukemic patients was determined by 3-(4,5-dimethylthiazol-2-yl)-2,5-diphenyl tetrazolium bromide (MTT) and trypan blue dye exclusion assay. Briefly, Cells (2 × 10^4^/250 μl/well) in log phase were seeded on 96-well tissue culture plates, incubated with freshly prepared WithaD (0-10 μM) for 24 and 48 hours at 37°C in a humidified atmosphere containing 5% CO_2_. After incubation, MTT (0.1 mg/well) was added, incubated for an additional 4 hr at 37°C. After plate centrifugation, the resultant pellet was dissolved in DMSO. Absorbance of the resultant formazon was measured at 550 nm using an plate reader (Multiskan Ex, Thermo electron corporation). Cell viability was also assessed by counting viable cells in each well in triplicate counting under light microscope. Peripheral blood mononuclear cells (PBMC_N_) from normal healthy individuals were exposed to WithaD under identical conditions. In parallel, a normal proliferative cell line (Vero, ATCC) was cultured in IMDM medium with 10% FBS and incubated with 10 μM of WithaD for 20 hr in similar condition. Each experiment was performed at least three times and in triplicates.

### Apoptosis assay

*In situ *DNA fragmentation was determined by TUNEL assay using APO-DIRECT kit according to manufacturer's instruction. Briefly, cells (1 × 10^6^) were washed with PBS, fixed in 1% paraformaldehyde and incubated in chilled 70% ethanol. After washing, cells were resuspended in staining solution containing TdT enzyme and FITC-dUTP and further incubated in PI/RNase staining buffer and analyzed by flow cytometer. For AnnexinV-7-AAD assay, cells (1 × 10^6^) were treated with WithaD and processed as reported earlier. Phosphatidylserine externalization was analyzed by double staining the cells with FITC-annexinV and 7-AAD reported earlier [23,24]. Cells were acquired and the data were analyzed by CellQuest Pro software (BD FACSCalibur).

### Flow cytometry

To check the status of the phosphorylation level of p38MAPK and JNK, cells were incubated for 0-4 hr with WithaD, washed and incubated with respective mAbs. Washed cells were subsequently incubated with PE-secondary antibody and analyzed by flow cytometer.

### Western blot analysis

Cells (1 × 10^6^) were treated WithaD and equal amount of protein were electrophoresed on SDS-PAGE (10-15%) and electrotransferred to nitrocellulose membranes. The membrane was then blocked by 2% PBS-BSA, probed with primary antibody overnight at 4°C, washed with PBS containing 0.1% Tween-20 and incubated with the appropriate horseradish peroxidase (HRP)-conjugated secondary antibody. The immunoreactive protein was identified either by the DAB-H_2_O_2 _system or ECL system [Pierce, USA, [25,26]].

### Ceramide Quantification

The ceramide level in cells was checked by intracellular immunostaining [27]. Briefly, cells were fixed and permeabilized with 1% formaldehyde/methanol in PBS for 10 min at room temperature. After washing, cells were incubated with anti-ceramide mAb, followed by FITC-labeled secondary antibody and the generation of ceramide was determined using flow cytometer. For blocking assay, cells were pretreated with Fumonisin B1 and/or GW4869 for 1 hr, followed by WithaD treatment for 2 hr and subsequently ceramide level were checked.

Ceramide content was also measured by diacylglycerol (DAG) kinase assay [28]. Neutral lipids were extracted as described previously [29]. Briefly, equal number of cells (5 × 10^6^) from treated and untreated were taken and cell pellets were disrupted by three cycles of freezing and thawing and homogenized by 30 s sonication at 10 watt. The aqueous pellets were extracted using chloroform/methanol (2:1, vol/vol) to obtain a final ratio of chloroform/methanol/water (8:4:2.4, v/v/v). After vortex and 15 min centrifugation at 1000 × g, the lower phase containing neutral glycolipids were recovered and evaporated under nitrogen. The samples were then resuspended in chloroform/methanol (2:1, v/v) and ceramide content was measured using [γ^32^P]-ATP (10 μCi) and 35 μg/ml recombinant E. coli DAG kinase at pH 6.5. Radioactive ceramide-1-phosphate was resolved by thin layer chromatography on silica gel 60 HPTLC plates using a solvent system of chloroform:methanol:acetic acid (65:15:5, v/v/v). Incorporated radioactivity was quantified using liquid scintillation counter.

In parallel, the samples were also loaded on a silica gel 60 HPTLC plate and similarly chromatographed along with standards (ceramide, SM). The plate was charred to visualize the bands.

### Sphingomyelinase Assay

N-SMase activity was measured using Amplex Red sphingomyelinase assay kit using manufacturer's protocol. Briefly, protein (50 μg) was diluted in assay buffer containing Tris-HCl (0.1 M), MgCl_2 _(10 mM), pH 7.4 and added (100 μl/well) to 96 well plate. Finally, the working solution (100 μl) containing alkaline phosphatase (8 U/ml), choline oxidase (0.2 U/ml), Amplex Red (0.1 mM) and HRP (2 U/ml) along with sphingomyelin (SM, 0.5 mM) was added to each well. The plate was then incubated for 30 min at 37°C in dark. The sequential breakdown of SM by N-SMase, alkaline phosphatase and choline oxidase produce H_2_O_2_. Finally, H_2_O_2_, in the presence of HRP, reacts with Amplex Red reagent to generate the specific fluorescent product, resorufin. The fluorescent intensity was measured immediately using Fluorescence plate Reader (Hitachi, Tokyo, Japan.) at 571/585 nm excitation/emission levels.

Acidic-SMase (A-SMase) activity was also measured using the same kit but in two-step process. Here, the cell lysate (100 μl/well) was diluted with sodium acetate (50 mM) buffer, pH 5.0. Subsequently, SM solution (5 mM, 10 μl) was added and incubated for 1 hr at 37°C for the generation of ceramide and phosphorycholine. Finally, the working solution (100 μl) was added to each well to react with phosphorylcholine for 45 min at 37°C. The H_2_O_2 _generated in this process was measured as stated above.

### Scanning Electron Microscopy (SEM)

Cells (5 × 10^6^) were treated with WithaD, incubated for 12 hr and then processed for morphological studies by SEM [30]. Briefly, cells were directly fixed overnight with glutaraldehyde (2.5%) solution in PBS, pH 7.2 and over night in osmium tetraoxide (1%) in the same buffer. The cell suspensions were dehydrated in an ethanol gradation. After drying with carbon dioxide by the critical point method and sputter coating with gold samples were examined on a SEM (VEGAII LSU, TESCAN, Czech Republic) and at least 15 microscopic fields were observed for each sample.

### Semi-quantitative reverse transcription-PCR (RT-PCR)

Total RNA was extracted using RNeasy mini kit (Qiagen, USA) and reverse transcribed into cDNA with random primer using Im-Pro-II-Reverse transcription system (Promega, USA) as reported earlier [29]. The PCR was carried out with specific primers (Table 1) in PTC-100 (MJ Research, MA, USA) and products were electrophoresed on agarose gel (1%) followed by staining with ethidium bromide and visualized under UV light. The signal intensity of the respective bands was measured by means of the Quantity one version 4.1.1 software using BIORAD image analysis system (CA, USA).

**Table 1:** Primer sequences

Primer	Primer sequence	Fragment Position	Primer Length (bp)	Tm (°C)
**Ceramide synthase (LASS2-001), size: 242 bp**				

**Sense Primer**	5'-TTCTTTGAGCTGTACGTGGCTA-3'	**549-571**	**22**	**60.07**

**Antisense primer**	5'-TCTCGGAACTTCTTGAGGAGAC-3'	**790-768**	**22**	**59. 99**

**Acid sphingomyelinae (A-SMase), size: 146 bp**				

**Sense Primer**	5'-GAGTAGAGGCCTAAGTTGAC-3'	**2181-2201**	**20**	**50.74**

**Antisense primer**	5'-GGAGTCCAAGTCTCTTATCT-3'	**2326-2346**	**20**	**49. 90**

**Neutral sphingomyelinase (SMPD2) size: 240 bp**				

**Sense Primer**	5'-ACAATCGACAGAAGGACATC-3'	**781-801**	**20**	**55.00**

**Antisense primer**	5'-AGTTCTTGGGTACCATTGTG-3'	**1020-1000**	**20**	**54. 99**

**Neutral sphingomyelinase (SMPD3) size: 296 bp**				

**Sense Primer**	5'-TGTTACCCCAACAAGTGTAACG-3'	**1681-1703**	**22**	**59.82**

**Antisense primer**	5'-TCGTCAGAGGAGCAGTTATCAA-3'	**1976-1954**	**22**	**60.02**

**Neutral sphingomyelinase (SMPD4) size: 177 bp**				

**Sense Primer**	5'-ATCCTGTGGAGTACAGCATC-3'	**864-884**	**20**	**55.04**

**Antisense primer**	5'-TGTGGTACAGAGGACTGTCA-3'	**1040-1020**	**20**	**54.89**

**β-actin, size:137 bp**				

**Sense Primer**	5'-CGACAGGATGCAGAAGGAG-3'	**1014-1032**	**19**	**62**

**Antisense primer**	5'-ACATCTGCTGGAAGGTGGA-3'	**1150-1132**	**19**	**58**

### RNAi mediated silencing

Three sets of sense and anti-sense stealth™ RNAi were used for SMPD3 (Table 2). Cells (8 × 10^5 ^cells/2 ml/well) were plated at 50% confluences in a six well plate in RPMI-1640 without antibiotics [31]. After 24 hr, lipofectamine 2000 (10 μl) and opti-MEM™ medium (250 μl) without serum were mixed and incubated for 5 min at room temperature. Separately, siRNA (150 nmol) was mixed with opti-MEM™ medium (250 μl) and incubated for 5 minute. Above two mixtures were combined and further incubated for 30 min at room temperature for complex formation and added to each well. After 8 hr of incubation in the CO_2 _incubator, the medium containing siRNA-lipofectamine 2000 complexes was replaced with fresh 10% FCS containing RPMI-1640 without antibiotics and the cells were further cultured as required.

**Table 2:** siRNA sequences

	Sequence	Designated as
**AJ250460- 1**		**siRNA 1**
	
Sense	5'-CGAGCAGCCACCAAAUUGAAAGAGC-3'	
	
Anti-sense	5'-GCUCUUUCAAUUUGGUGGCUGCUCGCU-3'	

**AJ250460- 2**		**siRNA 2**
	
Sense	5'-GGUGCAGGUGGGAAGCACACCUCAG-3'	
	
Anti-sense	5'-CUGAGGUGUGCUUCCCACCUGCACCUU-3'	

**AJ250460- 3**		**siRNA 3**
	
Sense	5'-CCACUGCCAACGUCUGCCUCCUGCC-3'	
	
Anti-sense	5'-GGCAGGAGGCAGACGUUGGCAGUGGCA-3'	

### Statistical analysis

All the results were expressed as the mean ± S.D. of data obtained from three separate experiments. All statistical analyses were evaluated using graph pad prism software (San Diego). Data were analyzed by the paired *t *test, and *P *< 0.05 was considered statistically significant.

## Results

### WithaD induced apoptosis in lymphoid and myloid leukemia cells

WithaD (Figure 1A) induced extensive anti-proliferative activity against both K562 and MOLT-4 cells as demonstrated by the total disintegration of cell morphology, a decrease in cell density (Figure 1B) and reduction in cell viability in a dose and time dependent manner (Figure 1C). We have checked the viability of normal lymphocytes in presence of WithaD (0-20 μM) for 48 hrs. WithaD did not show any adverse effect on normal lymphocytes (Figure 1D) as well as on a proliferative normal cell line Vero (Figure 1B). We further demonstrated 58.84% and 62.93% *in situ *nuclear DNA fragmentation in K562 and MOLT-4 cells treated with 1.5 μM and 0.5 μM WithaD respectively at 48 hr (Figure 1E).

### WithaD induced ceramide accumulation

**Figure 2:**
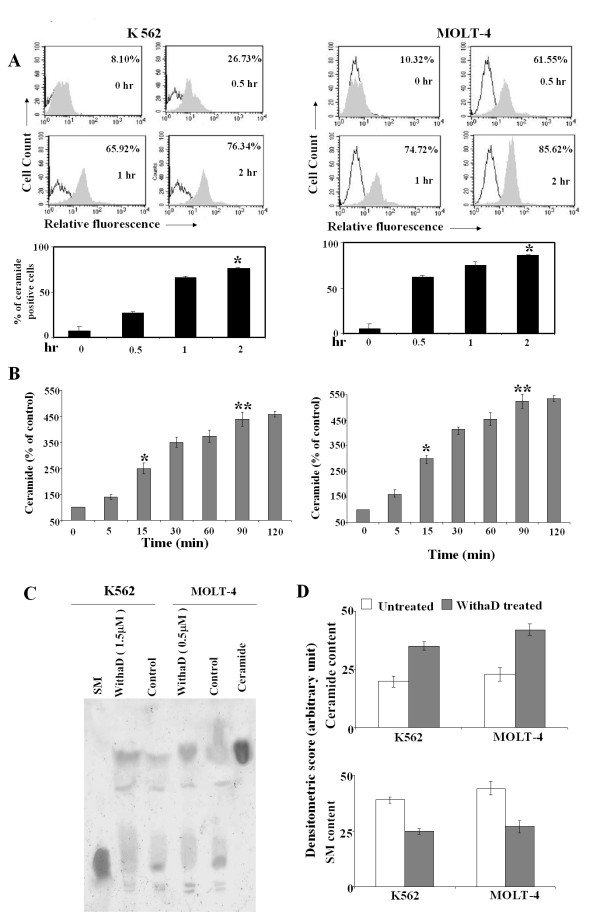
**WithaD induces ceramide production in K562 and MOLT-4 cells**. (A) Cells were treated with WithaD for 0-2 hr and the intracellular ceramide content were determined using anti-ceramide antibody by flow cytometer as described in Materials and methods. Representative histogram (top) and the percentages of positive cells (bottom) are shown. Results are the mean ± S.D. in duplicate in three independent experiments. Asterisk indicates statistically significant difference (P < 0.005) in 2 hr treated cells with respect to untreated cells. (B) The intracellular ceramide levels induced by WithaD in K562 and MOLT-4 cells were measured in the cell lipid extract by the diacylglycerol kinase assay as described in Materials and methods. Cells were incubated with 1.5 and 0.5 μM WithaD respectively at the indicated times. * represents statistical significant difference in ceramide level between 15 min and control, where P < 0.05. ** indicates significant difference in ceramide level between 90 min and control. Data are expressed as % of control values and are the mean ± S.D. of three different experiments performed in triplicate. (C) Level of ceramide and SM were determined in K562 and MOLT-4 cells separated in HPTLC Si 60 plates after 1 hr of WithaD treatment, along with standard ceramide and SM. This is a representative profile of three independent experiments. (D) Densitometric quantitation of ceramide and SM in lipid extracts from treated and untreated K562 and MOLT-4 cells. Data are mean ± SD of three independent experiments.

To ascertain whether ceramide has any potential role in WithaD induced cell death, we investigated the ceramide level using anti-ceramide antibody. K562 and MOLT-4 cells were separately treated with WithaD at different time points where we observed the increased ceramide level in a time-dependent manner that maximized at 2 hr (Figure 2A). Interestingly, MOLT-4 cells showed 61.55% even within 30 min of treatment whereas K562 cells showed only 26.73% positivity. In contrast, the endogenous ceramide levels were only 8-10% in untreated cells. Additionally, we measured the ceramide production by conventional DAG kinase assay. The results revealed almost 4-5 fold increase in ceramide production in K562 and MOLT-4 cells within 90 min of WithaD treatment (Figure 2B). For further confirmation of ceramide accumulation as a result of WithaD treatment, we isolated and separated neutral glycolipids on HPTLC that also revealed augmented ceramide level (Figure 2C). Densitometry of the TLC plates gives an approximate level of ceramide and SM. After WithaD treatment, ceramide was enhanced 1.75 fold in K562 and 1.83 fold in MOLT-4 cells as compared to respective untreated cells (Figure 2D).

### JNK and p38MAPK signals downstream of ceramide

**Figure 3:**
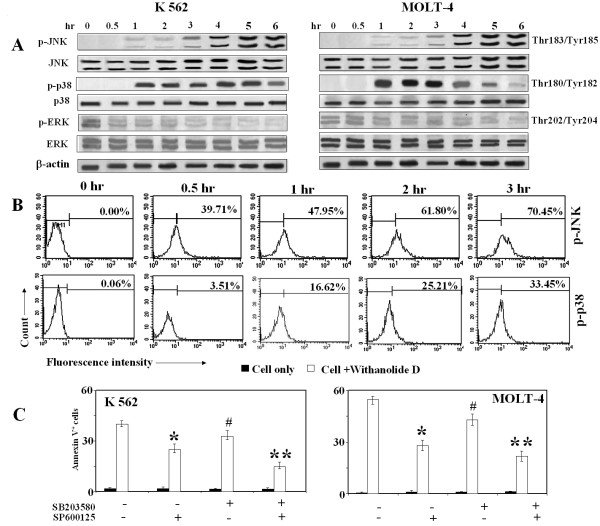
**Effect of WithaD on MAPKs in K562 and MOLT-4 cells**. (A) K562 and MOLT-4 cells were treated with WithaD at the indicated time and cell lysates were resolved in SDS-PAGE (10%). Western blot analysis showed higher level of phosphorylation of JNK and p38 whereas reduced level of p-ERK was observed in different time duration. (B) MOLT-4 cells were treated with WithaD at the indicated time and p-JNK^+ ^and p-p38^+ ^cells were analyzed by flow cytometry. (C) K562 and MOLT-4 cells were pretreated with SP600125 (10 μM) and SB203580 (10 μM) for 1 hr, then treated with withaD for another 48 hr and % of annexin V^+ ^cells were determined using flow cytometer. Results are the mean ± S.D. in duplicate in two independent experiments. *indicates statistically significant difference (*P *< 0.005) between SP600125 treated and untreated cells. **indicates statistically significant difference (*P *< 0.005) between SP600125 + SB203580 treated and untreated cells. # considered not significant difference (*P = *0.125) between SB203580 treated and untreated cells.

Ceramide activates multiple signaling pathways including the MAPKs. The members of MAPKs like ERK, p38MAPK and JNK/SAPK play the central role in survival and stress-induced cell death, in which ERK exerts opposing effects of p38MAPK and JNK/SAPK on apoptosis [32]. To investigate whether ERK, JNK and p38MAPK were involved, we monitored the effect of WithaD on K562 and MOLT-4 cells for 1-6 hr. Activation of JNK and p38MAPK were detected as early as 1 hr treatments of WithaD and persisted till 6 hr, whereas the reduced phosphorylation level of ERK was observed (Figure 3A). To further confirm the activation of both JNK and p38MAPK, we analyzed WithaD-treated MOLT-4 and K562 cells by flow cytometry. Interestingly, 39.71% p-JNK^+ ^and only 3.51% p-p38 MAPK^+ ^cells were observed in 1 hr in MOLT-4 cells (Figure 3B), whereas 23% p-JNK^+ ^and 3.1% p-p38 MAPK^+ ^cells were observed in K562. However after 3 hr, in MOLT-4 p-p38 MAPK^+ ^and p-JNK^+ ^cells were 33.45% and 70.45% respectively, whereas in K562, p-p38 MAPK^+ ^and p-JNK^+ ^cells were 31.15% and 58.34% respectively suggesting time dependent increase of both JNK and p38 MAPK in both the cell lines.

To ascertain the role of activated JNK and p38MAPK on WithaD-induced cell death, we used specific inhibitors of JNK (SP600125) and p38MAPK (SB203580) [33,34] and measured the rate of apoptosis after 48 hr of WithaD treatment. The addition of SP600125 significantly reduced the annexinV positivity from 40.92% to 25.83% in K562 whereas decrease of apoptosis by SB203580 from 40.92% to 32.88%, which is not significant (Figure 3C). Interestingly, when we treated the cells with both the inhibitors, apoptosis was further reduced to 14.72% for K562. Similar trend was observed in MOLT-4 cells. Therefore, our results suggest that JNK and p38MAPK might work cooperatively and amplify WithaD-induced apoptosis in leukemia cells.

### Activation of JNK and p38MAPK occurs through a common upstream regulator

**Figure 4:**
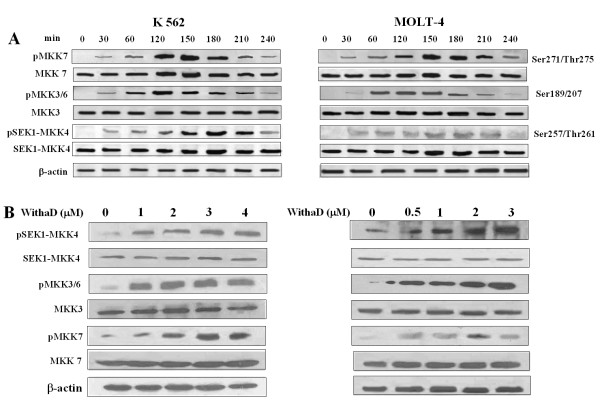
**WithaD activates MKK group of kinases upstream of p38 and JNK**. (A) K562 and MOLT-4 cells were treated with WithaD (1.5 and 0.5 μM respectively) for different time points. The level of phosphorylation of MKK4, MKK7 and MKK3/6 was determined in WithaD-treated cells by Western blot analysis using appropriate anti-MKK antibody. β-actin served as loading control. (B) The level of phosphorylation of MKK4, MKK7 and MKK3/6 was further determined after 2 hr treatment with different amount (0-4 μM) of WithaD as described above.

Cellular stress and anti-cancer agents activates both JNK and p38MAPK, but in a distinctively separate way [35]. MKK7 is a kinase that specifically activates JNK/SAPK whereas MKK3/6 serves as a specific activator of p38MAPK [36-38]. Here, in both the cell lines, MKK7 and MKK3/6 were activated within 30 min exposure of WithaD (Figure 4A). Interestingly, another MEKK homolog, SEK1/MKK4 was also activated within 30 min to a greater extent under similar treatment. Hence, our results might suggest that, SEK1/MKK4 was activated upstream of MKK3/MKK6 and MKK7 due to WithaD treatment. Activation of MKK4, MKK3/6 and MKK7 was also observed in a dose (0-4 μM) dependant manner with WithaD (Figure 4B).

### WithaD induces neutral-sphingomyelinase activation upstream of ceramide

**Figure 5:**
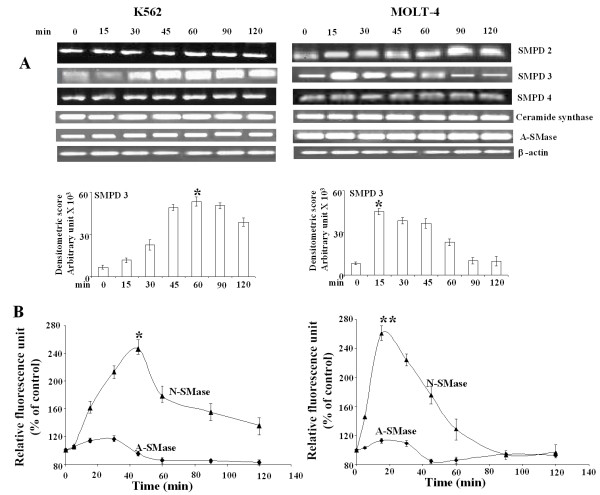
**WithaD induces N-SMase activation**. (A) RT-PCR analysis of N-SMases, ceramide synthase, and A-SMase. K562 and MOLT-4 cells were treated with WithaD (1.5 and 0.5 μM respectively) for 0-120 min. RNA was extracted from total cell lysate and RT-PCR was performed. The band intensity was measured. This is one representative of three independent experiments. *indicates statistically significant difference (*P *< 0.005) with respect to untreated cells. (B) Measurement of N-SMase activity using Amplex Red sphingomyelinase assay kit using manufacturer's protocol in K562 and MOLT-4 cells after WithaD (1.5 and 0.5 μM respectively) treatment. A-SMase activity was determined using the same kit by two-step SMase assay as described in Materials and methods. *indicates statistically significant difference (*P *< 0.005) between 45 min activity and 0 min in K562 cells whereas ** indicates significant difference (*P *< 0.005) in activity in MOLT-4 cells.

Intracellular ceramide may be generated either by *de novo *biosynthesis through ceramide synthase or by membrane sphingomyelin (SM) degradation catalyzed by sphingomyelinases [39,40]. Therefore to ascertain the source of ceramide production, we measured the mRNA level of ceramide synthase, A-SMase and N-SMase. Here, we observed a marked increase in N-SMase2 (SMPD3) mRNA level, which activated as early as 15 min in WithaD-treated MOLT-4 cells and gradually decreased after 45 min (Figure 5A). However, N-SMase 2 mRNA level activated within 30 min and persisted till 120 min in WithaD-treated K562 cells as revealed by densitometric analysis. In contrast, there were negligible changes in SMPD2 and SMPD4 in both leukemic and myeloid cells. No change in mRNA level of ceramide synthase and A-SMase was also observed under similar treatment.

We further measured the activity of both A-SMase and N-SMase after treatment of WithaD at different time points until 2 hr. Although N-SMase reached its maximal activity within 45-60 min in K562, the highest activity was observed even within 15 min in MOLT-4 cells, which subsequently decline within 2 hr (Figure 5B). The sharp decrease of N-SMase activity in MOLT-4 as compared to K562 (p53 null) suggested some relationship between p53 and N-SMase activation because p53 was known to regulate ceramide formation through N-SMase activation in glioma cells.

### N-SMase critically regulate ceramide production and activation of stress kinases

**Figure 6:**
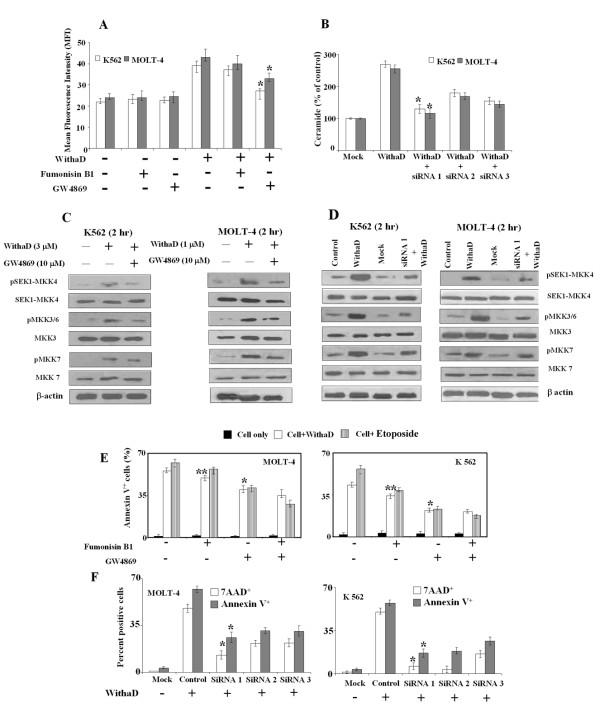
**Effect of Fumonisin B1, (GW4869) and N-SMase silencing on ceramide production, activation of MKK group of kinases and regulation of WithaD-mediated apoptosis**. (A) Effect of Fumonisin B1 (10 μM) and GW4869 (10 μM) on ceramide level induced by WithaD. Cells were pretreated with inhibitors for 1 hr; incubated further 1 hr with WithaD and ceramide levels were measured by FACS. Each column represented the mean ± S.D. in duplicate in three independent experiments. *indicates statistically significant difference (*P *< 0.005). (B) Effect of SMPD3 silencing in WithaD mediated ceramide accumulation. Cells were transfected with SMPD3 specific siRNAs as described in materials and methods. Ceramide level were measured by DAG kinase assay as described in Materials and methods. Results are the mean ± S.D. of two independent experiments. * indicates statistical significant difference *P *< 0.05. (C) and (D) Consequence of N-SMase inhibition by GW4869 and N-SMase silencing by siRNA1 on phosphorylation of MKK4, MKK3/6 and MKK7. K562 and MOLT-4 cells were pretreated with GW4869 (10 μM) for 1 hr and further incubated for 2 hr with 3 μM and 1 μM WithaD respectively. (E) Effect of Fumonisin B1 (10 μM) and GW4869 (10 μM) on cell death. Cells were pretreated separately with the inhibitors for 1 hr and after 48 hr apoptosis were measured. Each column represented the mean ± S.D. in duplicate in three independent experiments. * indicates statistically significant difference (*P *< 0.005). ** indicates difference (*p *= 0.1250). Etoposide was used as positive control. (F) Effect of SMPD3 silencing in WithaD treated cells. After 48 hr of transfection, apoptosis was measured. Results are representative of three independent experiments. * indicates statistical significant difference, where *P *< 0.05.

To confirm the role of N-SMase in ceramide production, we pretreated the cells separately with inhibitor of N-SMase (GW4869) and ceramide synthase (Fumonisin B1) [41,42] followed by WithaD treatment and measured the ceramide level. We observed that GW4869 mediated 30% reduction in K562 and 23% in MOLT-4 in ceramide level (Figure 6A). In contrast, fumonisin B_1 _treatment could not produce any significant change in ceramide level that ruled out the possibility of ceramide synthesis by *de novo *pathway rather than SM hydrolysis. Additionally, we had knockdown the N-SMase2 activity by silencing the SMPD3 gene using three sets of siRNA and observed that each siRNA oligonucleotide have potential silencing affect, where siRNA1 was most effective. As a result each set of siRNA potentially reduced ceramide production (Figure 6B). These results indicated that the source of accumulation of ceramide both in K562 and MOLT-4 cells could come from sphingomyelin hydrolysis rather than *de novo *synthesis suggesting the role of N-SMase in ceramide production.

Next, to address whether N-SMase directly regulates the activators of stress kinase JNK and p38MAPK, we have silenced the N-SMase2 with siRNA1 and separately also blocked it with its pharmacological inhibitor GW4869. Subsequently we measured the phosphorylation of MKK4, MKK7 and MKK3/6 (Figure 6C-D). Inhibition and silencing of N-SMase2 resulted in substantial decrease in phosphorylation of MKK4 and MKK3/6 in both the cell lines, suggesting the direct effect of N-SMase. Interestingly, the phosphorylation level of MKK7 was moderately effected by N-SMase knockdown or inhibition.

### N-SMase regulates WithaD induce cell death

To confirm that N-SMase plays a crucial role in WithaD mediated apoptosis, cells were pretreated with GW4869 with or without WithaD and further incubated for 48 hr. The % of annexinV^+ ^cells after GW4869 treatment significantly reduces from 55.63% to 39.67% in MOLT-4 and 43.25% to 21.89% in K562 cells (Figure 6E), whereas, fumonisin B_1 _treatment showed not significant (*p *= 0.1250) reduction of annexinV^+ ^cells. When we treated the cells with both the inhibitors, no substantial reduction of apoptosis was observed indicating that possibly N-SMase activation was one of the main factor responsible for WithaD-induced cell death. The results were similar as observed in etoposide treated cells, which was used as a positive control because it is a known chemotherapeutic agent that induces apoptosis via ceramide formation through N-SMase activation and not A-SMase [43].

The pivotal role of N-SMase was further confirmed by anti-sense knockdown assay, which showed that siRNA1 oligonucleotide eliminated 60-80% of apoptosis induced by WithaD as demonstrated by lower annexinV and 7-AAD positivity (Figure 6F).

### WithaD induced apoptosis in lymphoblasts of leukemia patients

**Figure 7:**
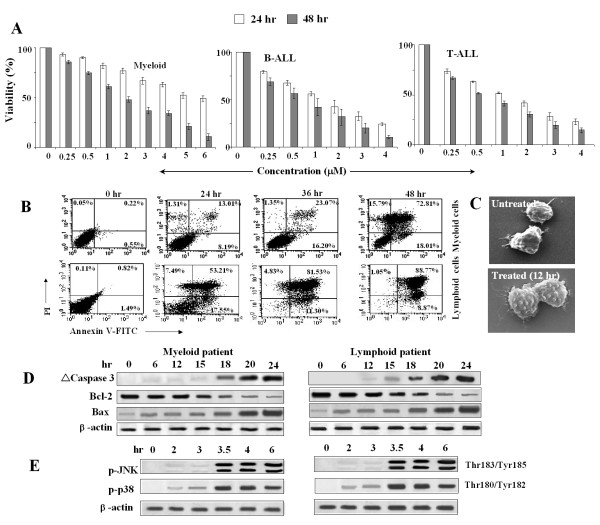
**WithaD induced apoptosis in primary cells from both myeloid and lymphoid patients**. (A) WithaD reduced the viability of the cells from pediatric myeloid, T- and B-ALL patients in 24 and 48 hr in a dose dependent manner as assessed by trypan blue dye exclusion test. (B) WithaD potentially induced apoptosis in primary cells from patients. WithaD treated primary cells from representative lymphoid and myeloid patients showed increased annexin V+/PI- (down right quadrant) and annexin V+/PI+ (upper right quadrant) cells by flow cytometry. (C) A representative SEM micrograph showing typical blebbing in cell membrane in untreated (×10000) and treated (×10000) lymphoblast. (D) Cell lysates of primary cells from patients were resolved in 10% SDS-PAGE. Western blot analysis showed the cleaved caspase 3, changes in Bcl-2 and Bax expression, after WithaD treatment in a time dependent manner. b-actin was used as loading control. (E) Cell lysates of primary cells from patients were resolved in 10% SDS-PAGE. Western blot analysis showed enhanced phosphorylation of JNK and p38 MAPK in response to WithaD treatment in a time dependent manner. β-actin was used as loading control.

To establish the *in vivo *condition, we have investigated the effect of WithaD on fresh leukemia cells of clinically confirmed B-(n = 10), T-(n = 5) ALL and myeloid (n = 7) patients. A dose dependent growth inhibition was observed after 24 and 48 hr exposure in both myeloid and lymphoid cells using trypan blue dye exclusion assay (Figure 7A). Treatment with WithaD for 48 hr resulted ~80% annexinV^+ ^lymphoblasts in these patients (Figure 7B). Results from representative lymphoid and myeloid patients were shown. The morphological analysis of patient cells with WithaD for 12 hr showed significant changes under SEM, whereas untreated cells remained unaltered (Figure 7C).

For further confirmation of the signaling pathway, we demonstrated that the anti- and pro-apoptotic protein level of Bcl-2 and Bax was changed in a time dependent manner, in patients' cells treated with WithaD as demonstrated by Western blot analysis. Subsequently, WithaD induces caspase 3 activation in these patients (Figure 7D). Additionally, WithaD-treated patients cells showed phosphorylation of JNK and p38MAPK in a time dependent manner (Figure 7E). All these pre-clinical studies using the patients' samples suggested that WithaD might have potential to kill the lymphoblasts *in vivo*.

## Discussion

Current investigations are giving new insight about the roles of sphingolipids in cellular regulation of growth suppression, apoptosis and signal transduction. Among the sphingolipids, ceramide has emerged as an important second signal mediator, having multiple cellular and biochemical targets. In particular, ceramide act as a potent cell death inducer and a specific cell growth suppressor. Recently, it has been thought that generation of ceramide is a universal feature of apoptosis [6,44]. Presently, a few drugs are available in combinatorial chemotherapy, which could generate ceramide. Therefore, to find pharmacological modulators that induce ceramide production in malignant cells by mounting a physiological response and induce senescence is on prime importance.

In the present study, we demonstrated rapid accumulation of endogenous ceramide in WithaD treated MOLT-4 cells. In fact, 61.55% cells were ceramide positive just in 30 mins. WithaD showed almost four fold increase in ceramide level within 2 hr in both the cell lines suggesting that ceramide plays a crucial role in WithaD-induced apoptosis. A number of phosphatase and kinases involved in stress signaling are activated/regulated by ceramide. Ceramide targets stress-activated protein kinases (SAPKs) such as thecc-jun N terminal kinases (JNKs), kinase suppressor of Ras (KSR), and the atypical protein kinase C (PKC) isoform, PKCζ [45,46]. Ceramide is also capable of activating protein phosphatases such as protein phosphatase 1 (PP1) and protein phosphatase 2A (PP2A) [47]. Among the vast targets of ceramide, the kinase signalling cascade was an important pathway in the transduction of apoptotic signals initiated by stress stimuli, and the main participant in this cascade were JNK and p38MAPK as well as their upstream kinases (MKKs) [48]. Extracellular stimuli activate MKK7 that serves as a specific activator of JNK/SAPK, whereas in a similar mechanism, MKK3/6 activates p38MAPK. However, besides MKK7 and MKK3/6, a dual specific threonine tyrosine protein kinase MKK4 known to phosphorylate and activates both JNK and p38MAPK [49].

In our study, we found that downstream of ceramide, both JNK and p38 were activated within 2-3 hr in response to WithaD. Also, within 60 min, MKK4, MKK3/6 and MKK7 were activated. Knockdown and inhibition of N-SMase reveal that Posphorylation of MKK4 and MKK3/6 were affected, whereas pMKK7 remain almost unaltered. These results suggested that besides N-SMase induced direct activation of MKK4 and MKK3/6, MKK7 was parallely activated. Inhibition of JNK and p38MAPK separately decreased WithaD-induced apoptosis. However, the combined treatment of SP600125 and SB203580 further reduced the annexinV^+ ^cells, which gives the plausible explanation that these two stress-related pathways worked cooperatively to amplify the pro-apoptotic signal.

Ceramide is a known important second signal effector molecule. Several lines of evidence indicate that under stressed condition, sphingomyelin turnover is induced resulting in its breakdown and increase in ceramide level. Intracellular ceramide is primarily generated, either by sphingomyelin hydrolysis via the action of sphingomyelinase or by *de novo *pathways involving ceramide synthase [6,35,50-52]. N-SMase and A-SMase have been demonstrated to involve the formation of ceramide in response to apoptotic inducers including chemotherapeutic agents [53,54]. On the contrary, sphingosine kinase (SHPK) is an enzyme that produces sphingosine 1-phosphate (S1P) from the ceramide breakdown product sphingosine, which help in survival. Therefore, these enzymes play crucial role determining cells fate. Interestingly, in leukemia, it was reported that SHPK1 was upregulated, whereas N-SMase level was decreased. So, either the decrease of N-SMase or increase in SHPK1 resulted in lower ceramide/SIP ratio, which helps in the survival of lymphoblasts [55]. Our result revealed an increase in intracellular ceramide content accompanying with SMPD3 activation as an early event of WithaD treatment, suggesting WithaD possibly hit the sphingomyelin hydrolysis by N-SMase 2 to generate ceramide, which may modify the ceramide/SIP ratio paves the way from survival to death. Furthermore, inhibition of N-SMase by GW4869 or silencing of SMPD3 by specific siRNAs protects the cells from the cell death suggesting the importance of N-SMase activation. Recently, Ito et al reported that three Sp1 motifs located between -148 and -42bp upstream of the first exon of N-SMase were important in its activation, at least by Daunorubicin induction [56]. However, the transcriptional regulation of N-SMase activation by WithaD was not clear and yet to be determined. Many studies suggested a connection between oxidative stress and N-SMase activation. In fact, early ROS production is a critical event in ceramide generation [57] and cell death. Therefore, we have investigated the production of ROS at early time point as maximal activation of N-Smase was observed within 2 hr exposure of WithaD. However, WithaD treatment did not produce ROS within 0-2 hr in both the cell lines under this experimental condition suggesting that WithaD mediated N-SMase activation was possibly ROS independent.

**Figure 8:**
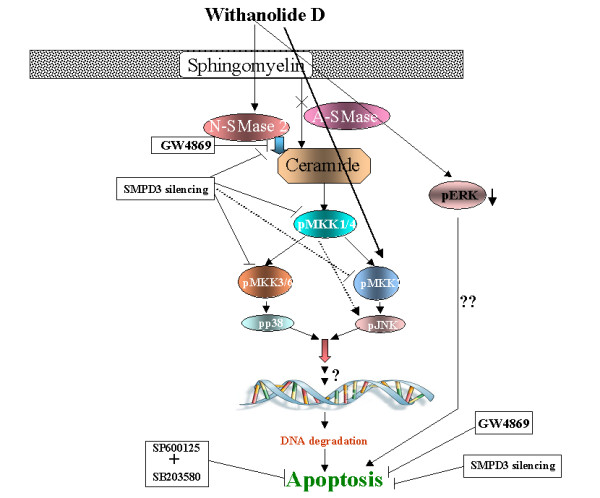
**Probable mechanism of WithaD induced apoptosis of K562 and MOLT-4**.

Natural products have received increasing attention for the search of novel cancer preventive and therapeutic agent. However, the pharmacological activity and molecular targets of most of the ancient treatment and their bioactive compounds are ignored and not well studied [58]. However, many naturally occurring plant products have the potential to target multiple signaling pathways. WithaD is isolated from a well known medicinal plant through cross-breeding of germplasm lines in high yields. To the best of our knowledge, pharmacological investigation of pure WithaD is totally absent, therefore, demanding such investigations. Recently, we have observed WithaD induced *in vitro *and *in vivo *cell death in leukemia (personal communication). Here we showed that (a) not only human myeloid (K562) and lymphoid (MOLT-4) cell lines but also primary cells from leukemia patients are highly sensitive to growth inhibition by WithaD in a dose and time dependant manner; (b) WithaD-mediated apoptosis was through the activation of N-SMase 2 and accumulation of ceramide content and (c) ceramide activate MKK group of proteins, leading to JNK and p38MAPK phosphorylation, resulting in their cooperative action to transduce the death signal. These results provide a basic mechanism which indicate that WithaD activate multiple signalling cascade to induce apoptosis in leukemia (Figure 8). These findings suggest that WithaD is a promising herbal compound that may be useful in future novel anti-leukemia strategies by targeting multiple pathways along with ceramide accumulation through N-SMase 2 activation.

## Abbreviations

WITHAD: Withanolide D; ALL: Acute lymphoblastic leukemia; PCD: programmed cell death; FACS: fluorescence activated cell sorter; mAb: monoclonal antibody; TUNEL: terminal deoxynucleotidyltransferase dUTP nick end labeling; JNK: c-Jun N terminal kinase; ERK: extracellular signal-regulated kinase; MAPK: mitogen activated protein kinase; SM: sphingomyelin; N-SMase: Neutral sphingomyelinase; A-SMase: Acid sphingomyelinase; SHPK: sphingosine kinase; S1P: sphingosine 1-phosphate.

## Patent

A novel chemotype of Ashwagandha (Withania somnifera) named 'NMITLI-135' that lacks withaferin-A and withanone and hyper accumulates withanolide D and glycowithanolide withanoside VI in leaf. (No. 187NF2007).

## Competing interests

The authors declare that they have no competing interests.

## Authors' contributions

SM performed the experiments, analysed and interpreted the experimental findings and drafted the manuscript. CM perfomed the TLCs. RS isolated the compound and provided for the study. SC supplied the blood sample of the patients and took part in analyzing patient data. Chitra Mandal supervises the experimental concept, designs, interpretation and helped to prepare the final version of manuscript. All authors read and approved the final manuscript.

## Supplementary Material

Additional file 1 **NMR-Spectral data of withanolide D**. Click here for file
