# Supplementary material for: Withanolide D induces apoptosis in leukemia by targeting the activation of neutral sphingomyelinase-ceramide cascade mediated by synergistic activation of c-Jun N-terminal kinase and p38 mitogen-activated protein kinase
Source: Mol Cancer. 2010 Sep 13;9:239. doi: 10.1186/1476-4598-9-239 (PMC2949798; doi:10.1186/1476-4598-9-239)
Supplement: Additional file 1 — NMR-Spectral data of withanolide D. [file 1476-4598-9-239-S1.DOC]

**Additional file 1: NMR-Spectral data of withanolide D**

**^1^H NMR (CDCl_3_, 300 MHz):** δ 6.20 (d, 1H, *J* = 10.0 Hz, H-2), 6.95 (dd, 1H, *J* = 10.0, 6.0 Hz, H-3), 3.75 (d, 1H, *J* = 6.0 Hz, H-4), 3.22 (br s, 1H, H-6), 0.85 (s, 3H, H-18), 1.40 (s, 3H, H-19), 1.25 (s, 3H, H-21), 4.18 (dd, 1H, *J* = 12.0, 4.0 Hz, H-22), 1.87 (s, 3H, H-27) and 1.94 (s, 3H, H-28).

**^13^C NMR (CDCl_3_, 75 MHz):** 202.0 (C, C-1), 132.3 (CH, C-2), 141.9 (CH, C-3), 69.9 (CH, C-4), 63.2 (C, C-5), 62.4 (CH, C-6), 31.5 (CH_2_, C-7), 30.2 (CH, C-8), 44.2 (CH, C-9), 47.2 (C, C-10), 21.9 (CH_2_, C-11), 39.7 (CH_2_, C-12), 42.0 (C, C-13), 56.6 (CH, C-14), 23.7 (CH_2_, C-15), 21.9 (CH_2_, C-16), 54.7 (CH, C-17), 13.4 (CH_3_, C-18), 17.2 (CH_3_, C-19), 75.0 (C, C-20), 20.4 (CH_3_, C-21), 80.9 (CH, C-22), 30.9 (CH_2_, C-23), 148.6 (C, C-24), 122.0 (C, C-25), 166.0 (C, C-26), 12.3 (CH_3_, C-27) and 20.7 (CH_3_, C-28).
